# Vitamin B6 reduces hippocampal apoptosis in experimental pneumococcal meningitis

**DOI:** 10.1186/1471-2334-13-393

**Published:** 2013-08-27

**Authors:** Denise C Zysset-Burri, Caroline L Bellac, Stephen L Leib, Matthias Wittwer

**Affiliations:** 1 Biology Division, Spiez Laboratory, Federal Office for Civil Protection, Austrasse, CH-3700, Spiez, Switzerland; 2 Institute of Parasitology University of Bern, Länggassstrasse 122, CH-3012, Bern, Switzerland; 3 Graduate School for Cellular and Biomedical Sciences, University of Bern, Bern, Switzerland; 4 Neuroinfection Laboratory, Institute for Infectious Diseases, University of Bern, Friedbühlstrasse 51, CH-3010, Bern, Switzerland

**Keywords:** Bacterial meningitis, *Streptococcus pneumoniae*, Kynurenine pathway, Vitamin B6

## Abstract

**Background:**

Bacterial meningitis caused by *Streptococcus pneumonia*e leads to death in up to 30% of patients and leaves up to half of the survivors with neurological sequelae. The inflammatory host reaction initiates the induction of the kynurenine pathway and contributes to hippocampal apoptosis, a form of brain damage that is associated with learning and memory deficits in experimental paradigms. Vitamin B6 is an enzymatic cofactor in the kynurenine pathway and may thus limit the accumulation of neurotoxic metabolites and preserve the cellular energy status.

The aim of this study in a pneumococcal meningitis model was to investigate the effect of vitamin B6 on hippocampal apoptosis by histomorphology, by transcriptomics and by measurement of cellular nicotine amide adenine dinucleotide content.

**Methods and results:**

Eleven day old Wistar rats were infected with 1x10^6^ cfu/ml of *S*. *pneumoniae* and randomized for treatment with vitamin B6 or saline as controls. Vitamin B6 led to a significant (p > 0.02) reduction of hippocampal apoptosis. According to functional annotation based clustering, vitamin B6 led to down-regulation of genes involved in processes of inflammatory response, while genes encoding for processes related to circadian rhythm, neuronal signaling and apoptotic cell death were mostly up-regulated.

**Conclusions:**

Our results provide evidence that attenuation of apoptosis by vitamin B6 is multi-factorial including down-modulation of inflammation, up-regulation of the neuroprotective brain-derived neurotrophic factor and prevention of the exhaustion of cellular energy stores. The neuroprotective effect identifies vitamin B6 as a potential target for the development of strategies to attenuate brain injury in bacterial meningitis.

## Background

Bacterial meningitis (BM) caused by *S*. *pneumoniae* is a life-threatening disease associated with high mortality and morbidity rates. In spite of effective antimicrobial therapy and intensive care, about 50% of survivors suffer from long-term sequelae, including hearing loss, neurofunctional problems, seizure disorders, sensory-motor deficits, and persisting learning and memory difficulties [1-3].

Two pathophysiologically different forms of brain injury, namely hippocampal apoptosis and cortical necrosis, have been demonstrated in patients [4] and in corresponding experimental animal models of BM. Damage to the hippocampal formation has been associated with learning and memory impairments [3,5].

**Figure 1:**
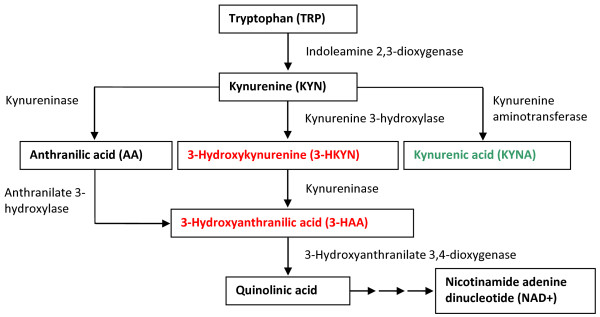
**Schematic of the kynurenine pathway in the rat brain.** Tryptophan is metabolized over multiple steps into quinolinic acid, finally resulting in *de novo* synthesis of NAD+. Several neuroactive intermediates are included in this pathway: neuroprotective kynurenic acid, neurotoxic 3-hydroxykynurenine and neurotoxic 3-hydroxyanthranilic acid. Neurotoxic intermediates are written in red, neuroprotective ones in green [6,7].

Inflammatory conditions in the brain induce tryptophan (TRP) degradation through the kynurenine (KYN) pathway, resulting in several neuroactive metabolites which can be both, neurotoxic or neuroprotective (Figure 1). The KYN pathway may be involved in the mechanisms leading to brain damage associated with inflammatory brain diseases, such as multiple sclerosis or cerebral malaria [6,7]. The pathophysiology of pneumococcal meningitis is initiated by activation of the immune system of the host, leading to the induction of metabolic pathways in the brain [6]. Increased TRP degradation caused by the activation of the KYN pathway may also be involved in the processes that result in neuronal damage observed in pneumococcal meningitis [2,6,8]. The neurotoxic effect of the intermediates 3-hydroxykynurenine and 3-hydroxyanthanilic acid involves the generation of superoxide and hydrogen peroxide that contribute to oxidative processes implicated in the pathophysiology of meningitis. In contrast, neuroprotective kynurenic acid (KYNA), an antagonist of the excitotoxic *N*-methyl-D-aspartate (NMDA) receptor, protects from excitotoxic brain damage in experimental BM [6]. Furthermore, the catabolism of TRP over the KYN pathway is the exclusive *de novo* synthesis pathway for nicotine amide adenine dinucleotide (NAD+) in eukaryotic cells [6]. NAD+ fuels the poly(adenosine 5′-diphosphate (ADP)-ribose) polymerase whose over-activation during neuro-inflammatory diseases may deplete intracellular NAD+ levels and thus, resulting in necrotic cell death [9]. Therefore, the KYN pathway induced in pneumococcal meningitis may influence the fate of neuronal tissue over NAD+ supply [6,9].

Pyridoxal 5′-phosphate, the active form of vitamin B6, optimizes the substrate flux in the KYN pathway by acting as cofactor for two key enzymes, KYN aminotransferase and kynureninase [10]. Administration of vitamin B6 may attenuate neuronal cell death in BM by preventing both, the accumulation of neurotoxic intermediates of the KYN pathway and cellular energy depletion by enhancing the *de novo* synthesis of NAD+.

In the present study, we evaluated the mode of action of vitamin B6 by microarrays. We interpreted the transcriptomic data using biological system based analysis rather than a “gene by gene” approach. The Gene Ontology (GO) [11] and the Kyoto Encyclopedia of Genes and Genomes (KEGG) pathway [12] database provide a basis for grouping genes according to their molecular functions, biologic processes and cellular components, and their involvement in concordant cellular pathways, respectively.

Histopathological analysis showed that vitamin B6 significantly reduced hippocampal apoptosis in pneumococcal meningitis. Furthermore, based on fluorescence measurements of hippocampal NAD+ levels, an effect of vitamin B6 in preserving cellular energy stores was found.

## Methods

### Ethics statement

All animal studies were approved by the Animal Care and Experimentation Committee of the Canton of Bern, Switzerland (Nr. 26/07), and followed the Swiss national guidelines for the performance of animal experiments.

### Model of experimental pneumococcal meningitis

We used an established model of experimental pneumococcal meningitis in infant rats [13]. On postnatal day 11, Wistar rats (n = 28) were infected by intracisternal injection of 10μl of saline solution containing 1 × 10^6^ cfu/ml of *S*. *pneumoniae* (serotype 3). At time of infection, animals (n = 14) received 360μl of vitamin B6 subcutaneously (s.c.; 600mg/kg; Streuli, Uznach, Switzerland). Placebo-treated animals (n = 14) were injected s.c. with 360 μl of 0.85% NaCl. Eighteen hours after infection, all animals were treated s.c. with 100 mg/kg of the antibiotic ceftriaxone (Roche Pharma, Reinach, Switzerland) and a second dose of vitamin B6 or 0.85% NaCl was administered. At the same time point, infection was documented by quantitative culture of 5 μl of cerebrospinal fluid (CSF) and all rats were weighed and clinically assessed using the following score system: 1 for comatose animals, 2 for rats that do not turn upright after positioning on the back, 3 for animals that turn within 30 s, 4 for animals that turn within less than 5 s and 5 for rats with normal activity [13]. Twenty four hours after infection, the rats were sacrificed by an overdose of intraperitoneal (i.p.) pentobarbital (100 mg/kg, Esconarkon, Streuli & Co. AG, Uznach, Switzerland).

For NAD+ measurements, Wistar rats (n = 15) were infected by intracisternal injection of 10 μl of 1×10^6^ cfu/ml of *S*. *pneumoniae*. At time of infection, animals were randomized for treatment with vitamin B6 (600 mg/kg s.c. 0 and 18 h p.i., n = 6) or an equal volume (360 μl) of 0.85% NaCl (s.c., n = 9). Three saline-treated rats were sacrificed at the same time point (0h p.i.). The antibiotic therapy was started 18 h post-infection (100 mg/kg ceftriaxone, s.c.). At the same time point, 3 vitamin B6- and 3 saline-treated rats were sacrificed (18 h p.i.), and a second application of vitamin B6 and 0.85% NaCl, respectively, were administered to the remaining 6 animals. These animals were sacrificed 24 h after infection (24 h p.i.).

### Tissue processing

Immediately after sacrifice, the animals were perfused via the left cardiac ventricle with 30 ml of RNAse-free ice-cold phosphate buffered saline (PBS). The brains were dissected followed by removal of the meninges and segmentation of the brains into the 2 hemispheres. The right hemisphere was fixed in 4% paraformaldehyde (Grogg, Stettlen-Deisswil, Switzerland) in PBS for 3 days at 4°C and then cryo-protected in 18% sucrose at 4°C until further processing for histopathological assessment of brain injury. From the left hemisphere the hippocampus was dissected in ice-cold PBS, stored in RNAlater® (Ambion Europe Ltd., Huntingdon, UK) for 1 day at 4°C and subsequently at −80°C until isolation of RNA. For NAD+ measurements, the hippocampus of the left hemisphere was frozen on dry ice and stored at −80°C.

### Histopathology

To assess the brain damage caused by BM the brains were analyzed histomorphologically. The cryo-protected brains were frozen in 2-methylbutane (−50°C), and from each animal four 45 μm cryo-sections of the dentate gyrus were cut using a Cryostat (Leica CM1850 cryostat) and transferred onto a gelatin/chrom alum-coated glass slide. The slides were put in Xylol, hydrated, Nissl stained with cresyl violet, dehydrated and mounted with Entellan® (Merck, Darmstadt, Germany). The amount of apoptotic cells in the dentate gyrus of the hippocampus and of the extent of damage to the cerebral cortex were evaluated using bright-field microscopy. Neurons of the dentate granule cell layer with morphological changes characteristic for apoptosis (condensed, fragmented nuclei and/or apoptotic bodies) were counted in 3 visual fields (400× magnification) in each of the 2 blades of the dentate gyrus. An average score per animal was calculated from all sections evaluated, applying the following scoring system: 0–5 cells = 0, 6–20 cells = 1 and >20 cells = 2 [14]. The cortical damage was assessed as the amount of damage of the total volume of the cortex as previously reported [15].

### RNA isolation, quality control and chip hybridization

From tissue samples of the hippocampus total RNA was isolated using the magnetic beads based EZ1 RNA Universal Tissue Kit (Qiagen, Basel, Switzerland) and EZ1 BioRobot (Qiagen). Tissue stabilized in RNAlater® was mixed with 750 μl QIAzol® Lysis reagent. Samples were immediately homogenized by a rotor-stator homogenizer (TissueRuptor®, Qiagen). After incubation for 5 min at room temperature, 150 μl chloroform (Grogg) was added to the homogenized tissue samples. A centrifuging step for 15 min at 4°C and 12’000 rpm resulted in the separation of the sample into 3 phases. 300 μl of the upper phase containing RNA was used as starting material for RNA isolation using the EZ1 BioRobot, following the manufacturer’s protocol.

Quantification of RNA was performed on the Agilent 2100 Bioanalyzer platform (RNA 6000 Nano, Agilent Technologies, Waldbronn, Germany) and validated on the NanoDrop® (NanoDrop, Wilmington, USA) device.

From 28 histopathologically evaluated rat brains with evidence for apoptosis, RNA extracts from 5 vitamin B6- and 5 saline-treated animals were selected randomly for array hybridization. Chip hybridization was performed in cooperation with the Lausanne DNA Array Facility (University of Lausanne, Switzerland). Double-stranded cDNAs were synthesized from 100ng of total RNA using T7 promoter-(N) 6 primers (Affymetrix, Santa Clara, CA) and the Whole Target Transcript cDNA synthesis kit (Affymetrix). Quantification and quality control of cDNA was performed by NanoDrop® and Agilent 2100 Bioanalyzer platform, respectively. Three microgram of fragmented, biotinylated cDNA was hybridized in a Hybe Oven (GeneChip® 640) overnight onto commercially available GeneChip® Rat Gene 1.0 ST Array (Affymetrix) containing over 27’000 rat genes. The hybridized samples were stained with streptavidin phycoerythrin and the signal was amplified by a biotinylated anti-streptavidin antibody. Washing, staining and amplification were performed in an Affymetrix GeneChip® Fluidics Station 450. The components required for these steps were provided by the GeneChip® Hybridization, Wash, and Stain kit (Affymetrix). Microarrays were scanned in an Affymetrix GeneArray® scanner 3000. Resulting image files served as basis for the calculation of signal intensities with the Affymetrix GeneChip® Operating Software (GCOS).

### Datamining

All data is MIAME compliant and has been deposited in the ArrayExpress database of the European Bioinformatics Institute (http://www.ebi.ac.uk/arrayexpress, accession number E-MEXP-3555).

Chip data analysis was carried out on the R platform for statistical programming using packages from the Bioconductor project [16]. Because of the asymmetric distribution of microarray data, expression values were log2 transformed. Background correction, quantile normalization and probe set summary (robust regression, only perfect matches) were performed with non linear methods based on the robust multi average (RMA) function of the Bioconductor affy package [17]. Chip quality control was explorative evaluated using box plots of the raw log scale intensities and MA-plots visualizing signal intensity dependent effects on the log-ratios (affy package).

To reduce the number of hypothesis to be tested in the adjacent significance tests, genes were filtered based on the following criteria: all genes that were expressed under the estimated background intensity of 2^6^ fluorescent units on at least 4 of the 10 arrays and genes with an interquantile range of less than 0.001 were excluded. Differentially expressed genes were identified by using the linear models for microarray data (limma) package [18] which implements a moderated t-statistic for significance testing. The type 1 error rate was adjusted to 1% using the Benjamini-Hochberg false discovery rate algorithm [19]. Genes which had cross-hybridized on the chip or which had no annotation in any existing data bank were excluded from further analysis.

The transcriptomic data were evaluated by the functional annotation clustering tool of DAVID (Database for Annotation, Visualization and Integrated Discovery) bioinformatics (http://david.abcc.ncifcrf.gov/) for GO statistics [11] and by biological system based analysis using the KEGG pathway database (http://www.genome.jp/kegg) for pathway analysis [12].

### Chip validation by real time PCR

cDNA was synthesized from 1.5 μg of total RNA using the High-Capacity cDNA Reverse Transcription Kit (Applied Biosystems, Foster City, CA), according to the manufacturer’s protocol. The cDNA samples were diluted 1:5 with RNAse-free water and aliquots were stored at −20°C. Real time PCR was performed using the QuantiFast Probe PCR kit from Qiagen (composed of Hot Star Taq Plus DNA polymerase and dNTP mix in PCR buffer) and TaqMan® Gene Expression Assays (Applied Biosystems).

All reactions were carried out as duplicates. Template cDNA was amplified with the Rotor-Gene Q platform (Corbet RESEARCH) operating with the Run on Software version “Rotor-Gene 1.7.87”.

The primers used for this PCR and their reference sequences (RefSeq) as well as their ordering numbers of Applied Biosystems are listened in Table 1. The delta Ct values were calculated based on normalization to the housekeeping gene *ribosomal protein L24* (Rpl24).

**Table 1:** **TaqMan**® **gene expression assays** (**Applied Biosystems**)

**Name**	**RefSeq**	**Assay number**
Brain-derived neurotrophic factor (BDNF)	NM_012513.3	Rn01484924_m1
Neuronal PAS domain protein 4 (Npas4)	NM_153626.1	Rn00596522_m1
Lysozyme 2 (Lyz2)	NM_012771.2	Rn00562794_m1
Platelet-activating factor acetylhydrolase 2 (Pafah2)	NM_177932.2	Rn00710058_m1
Nuclear receptor subfamily 4, group A, member 1 (Nr4a1)	NM_024388.1	Rn01533237_m1
Ribosomal protein L24 (Rpl24)	NM_001007637.1	Rn01455518_g1

The genes selected for chip validation by real time PCR and their corresponding reference sequences (RefSeq) and ordering numbers of Applied Biosystems. Rpl24 was used as housekeeping gene.

### Hippocampal NAD+ levels

For assessment of cellular energy status NAD+ levels were measured in hippocampal tissue from rats with BM treated with vitamin B6 or saline at 0, 18 and 24 hours after infection (n=3 for each experimental group and time).

Frozen dissected hippocampi were homogenized 1:10 (wt/vol) in ice-cold assay buffer (50 mM Tris and 2 mM MgCl2, pH 8.0) and 50 μl of the homogenates were transferred into a 96-well fluorescence plate. NAD+ was quantified according to the method of Putt and Hegenrother [20]. The plate was read on a SpectraMax Plus (Molecular Devices, Sunnyvale, CA) with an excitation of 360 nm and an emission of 445 nm, and values were plotted against a NAD+ calibration curve (Sigma, St. Louis, MO).

## Results

### Clinical parameters of meningitis

By 18 h after infection, all rats infected with *S*. *pneumoniae* had meningitis, as evidenced by positive bacterial titers in the CSF (log10 6.8 - log10 8.0 cfu/ml) and reduced weight gain. Between 18 h and 24 h post-infection, the animals treated with vitamin B6 lost 0.50 ± 0.04 g of body weight, whereas the weight of placebo-treated animals was reduced by 0.74 ± 0.02 g (p < 0.07, unpaired t test).

### Apoptotic cells in the dentate gyrus

**Figure 2:**
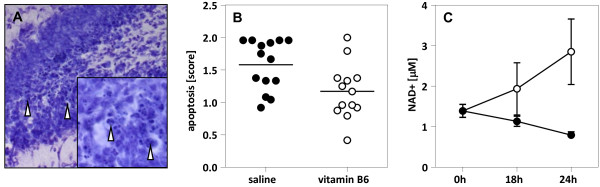
**Apoptosis in the rat hippocampal dentate gyrus after infection with *S*. *pneumoniae*. A**: Histomorphological analysis show neurons forming apoptotic bodies (arrowhead), a morphological feature characterisitc for programmed cell death and evenly distributed over the lower and upper blade of the hippocampal dentate gyrus (Cresyl violet, original magnification 20× and 40× (insert)). **B**: The mean score of apoptotic cells (represented by the horizontal lines) in the vitamin B6-treated group is significantly smaller than the score of placebo-treated rats (unpaired t test, p > 0.02). **C**: Hippocampal NAD+ levels were measured at 3 different time points after infection. The amount of NAD+ in placebo-treated rats (solid circles; vertical bars show standard deviation) gradually decreases over time as an index of depletion of cellular energy stores in the hippocampus of infected rats. In contrast, the NAD+ level of rats treated with vitamin B6 (bare circles) rises from the time of infection to 24 h post-infection. Statistical analysis was performed using GraphPad Prism 4 (GraphPad Software, San Diego California, USA).

Infection with *S*. *pneumoniae* caused apoptosis in the subgranular zone of the dentate gyrus as reported previously (Figure 2A) [21]. At 24 h post-infection representing the acute phase of the disease, the mean score of apoptotic neurons was 1.6 ± 0.1 in saline-treated animals. In animals treated with vitamin B6 the apoptotic damage score was significantly lower (p < 0.02, unpaired t test) with a mean score of apoptotic cells of 1.2 ± 0.1 (Figure 2B). In contrast, vitamin B6 treatment had no significant effect on cortical damage (data not shown).

### Microarrays

**Figure 3:**
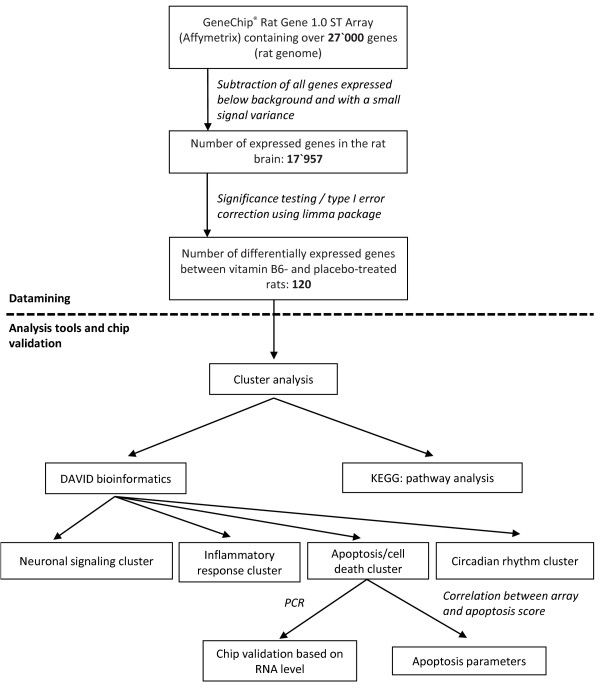
**Data analysis flowchart.** Overview of the workflow of data analysis chosen for the present work. Analysis was carried out on the R platform for statistical programming, cluster analysis was performed according to DAVID bioinformatics and pathway analysis was based on KEGG.

An overview of the workflow of microarray analysis chosen for this work is represented in Figure 3.

From over 27’000 rat genes represented on the chip, 17’957 genes were expressed over background level in the hippocampus of rat brains. After significance testing, the expression of 120 annotated genes was found to be influenced by vitamin B6 treatment (see Additional file 1: Table S1).

### Cluster analysis

**Figure 4:**
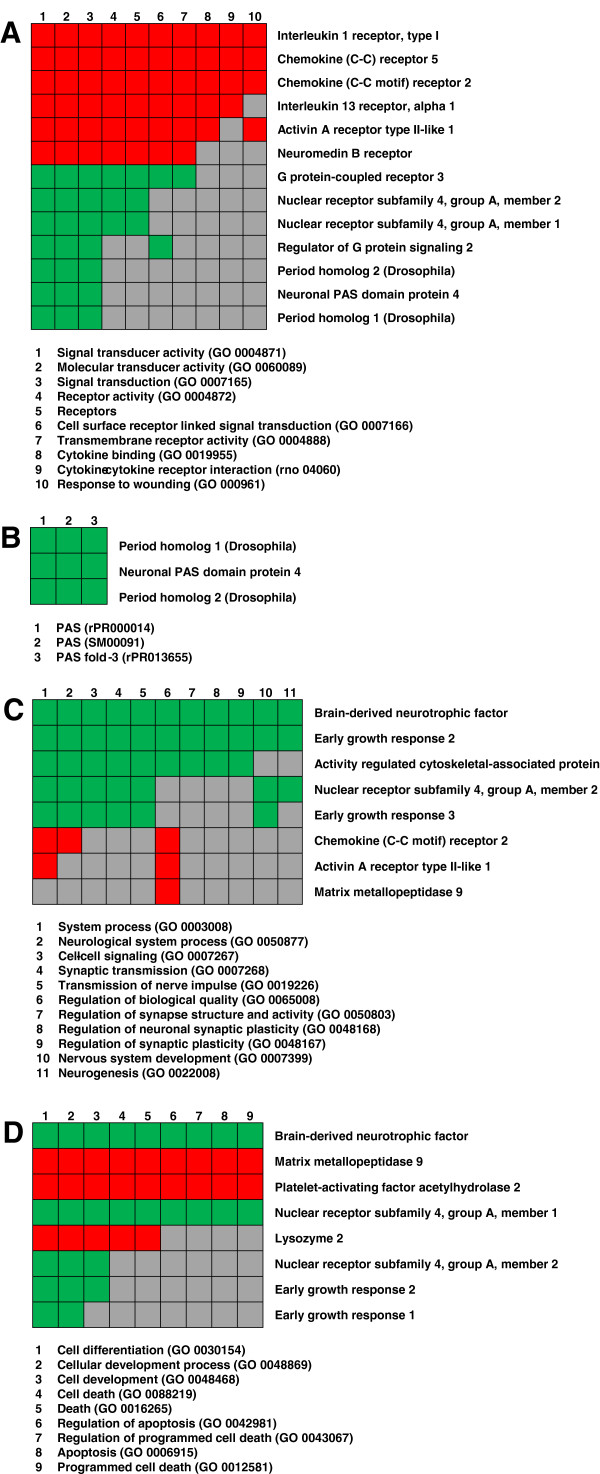
**Functional annotation cluster.** The 120 significantly expressed genes were clustered into functionally related groups such as inflammatory response **(A)**, circadian rhythm **(B)**, neuronal signaling **(C)** and apoptosis / cell death **(D)** based on DAVID bioinformatics. The genes belonging to the individual clusters are listened on the right side. The suitable functions with the corresponding GO terms are mentioned below the clusters. Green marked squares state that the gene is up-regulated in that GO term, red marked squares stand for down-regulated genes, and grey filled squares mean that the gene is not involved in that process.

Functional annotation clustering of significantly regulated genes by DAVID bioinformatics resulted in clusters of genes with possible roles in the mode of action of vitamin B6. The corresponding GO terms found to be overrepresented in our gene list belong to processes of the inflammatory response, circadian rhythm, neuronal signaling and apoptotic cell death (Figure 4A – D).

Upon vitamin B6 treatment many genes involved in processes of the inflammatory response (Figure 4A) were down-regulated such as pro-inflammatory cytokines and chemokines. In contrast, the majority of genes involved in processes of the circadian rhythm (Figure 4B) as well as of neuronal signaling (Figure 4C) and apoptosis (Figure 4D) were up-regulated.

A valuable tool to assign a set of genes to cellular pathways is the KEGG pathway database. The pathways over-represented in our set of genes were associated to the highly conserved mitogen-activated protein kinase (MAPK) cascade (see Additional file 2: Figure S1) as well as to the circadian rhythm (see Additional file 3: Figure S2). The MAPK pathway is involved in various cellular functions including inflammatory processes (e.g. down-stream signaling of IL-1) and neuronal signaling (e.g. via brain-derived neurotrophic factor), both processes also found by DAVID bioinformatics.

### Real time PCR

To validate the microarray data by an independent second method the expression levels of 5 genes (Table 1) were assessed by real time PCR. These genes were selected due to their putative importance in the mode of action of vitamin B6 in reducing hippocampal apoptosis, i.e. deriving from the apoptosis/cell death cluster, and because their expression levels span a wide signal range of the microarray.

The Pearson correlation between real time PCR and microarray data was r = 0.962 with a significant p value of 0.009.

### Correlation between RNA expression level and apoptosis score

**Figure 5:**
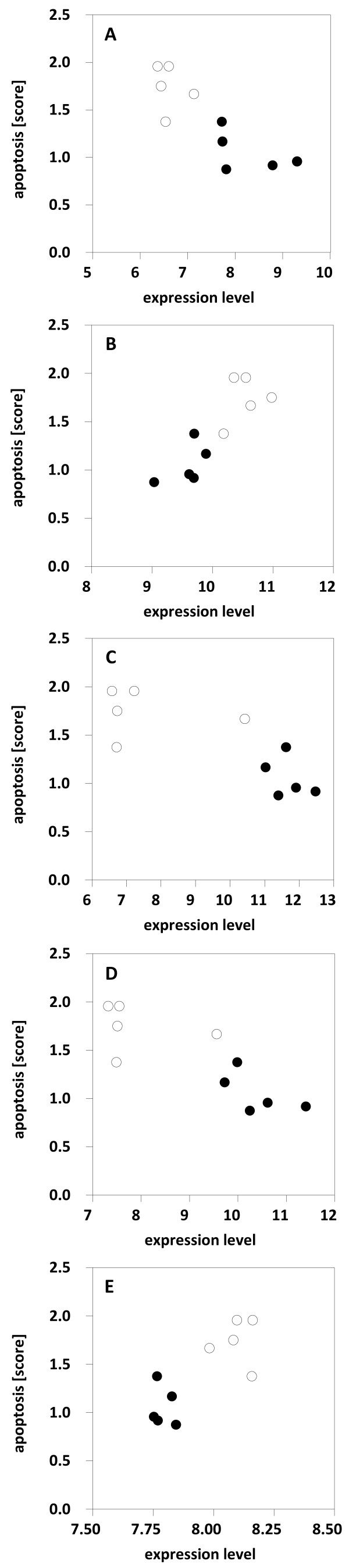
**Pearson correlation between RNA expression level and apoptosis score.** RNA expression data of individual rats are based on chip analysis, the apoptosis score is the mean value of apoptotic cells of corresponding rat brains. Expression levels of up-regulated genes such as BDNF **(A)** (Pearson correlation p = 0.002), Npas4 **(C)** (p = 0.005) and Nr4a1 **(D)** (p = 0.003) negatively correlate with apoptosis score, e.g. the less transcript, the more apoptotic cells. In contrast, expression levels of down-regulated genes such as Lyz 2 **(B)** (p < 0.001) and Pafah2 **(E)** (p = 0.005) postitively correlate with apoptosis score. Samples from vitamin B6-treated rats (bare circles) are arranged separately from samples of animals treated with saline (solid circles).

Pearson correlation between RNA expression levels of selected genes and apoptosis score was highly significant with p values between < 0.001 and 0.005 (Figure 5). The transcripts chosen for the correlation analysis were the same as assessed by real time PCR.

Due to these highly significant correlations between expression level and apoptosis score, the selected genes are candidates for apoptosis markers, although the time point is late (24 h post-infection) in regard to the notion that pro- and anti-apoptotic factors change at 12-16 h after infection [22]. However, further experiments are needed to determine the application of these genes as potential apoptosis markers.

### NAD+ levels in hippocampal tissue

Hippocampal NAD+ levels of rats with BM dropped after infection, indicating a decrease of cellular energy in the course of the disease. In contrast to the placebo group, the amount of NAD+ in the hippocampus of rats treated with vitamin B6 increased during the same time period (Figure 2C). Thus, an effect of vitamin B6 to preserve cellular energy stores, likely by optimizing the substrate flux in the kynurenine pathway, was found during the acute phase of BM when hippocampal apoptosis develops.

## Discussion

Apoptosis of cells in the hippocampal dentate gyrus is a characteristic form of brain damage in BM [3,21]. In experimental models an association of injury to the dentate gyrus with learning and memory deficits has been shown [5]. The present study demonstrates that treatment with vitamin B6 reduces the number of apoptotic cells in the hippocampal dentate gyrus. We investigated the mechanisms underlying this neuroprotective effect by studying the influence of vitamin B6 on the transcriptome and on cellular energy stores.

In the model used, hippocampal apoptosis starts to occur during the acute phase of BM (18-24 h) with a peak in the sub-acute phase at 36 h, and reaches control levels in the late phase of the disease (46-72 h). Recent studies in our lab showed that in the acute phase of BM, genes that are significantly expressed in the hippocampus and cortex are mainly involved in processes of the immune and inflammatory response [23]. The innate immune system has a predominant role in immune defense in the otherwise immune privileged brain tissue. However, neurological complications secondary to BM [3] suggest that the host defense mechanisms are inefficient in eliminating the pathogen and furthermore, that the host inflammatory reactions contribute considerably to the brain damage observed in the disease. As shown previously, the major events on transcriptional level concerning the regulation of the host immune response occur within the first 3 days after infection [23].

A promising candidate for pre-treatment of BM is vitamin B6 administered during the acute phase of the disease. A prerequisite for the application of a therapeutic target is the understanding of the processes leading to the desired effects, in the case of vitamin B6 to a reduction of hippocampal apoptosis. Thus, in the present study, we focused on the mechanisms that take place as a consequence of vitamin B6 treatment in the acute phase of BM. The time point for the second application of vitamin B6 (18 h post-infection) and the endpoint at 24 h post-infection were chosen during the acute phase of BM according to recent findings which define a time window for therapeutic interventions during this phase of the disease.

In order to handle the huge data mass resulting from a microarray study, we clustered the significantly regulated transcripts according to their involvement in given biological processes (Figure 4).

### Inflammatory response

The first step in immune activation is the recognition of bacterial products by pattern recognition receptors, such as Toll-like receptors (TLRs), expressed on resident cells of the central nervous system (CNS) [3]. Experimental studies showed that primary mouse microglial cells, the resident macrophages of the CNS, are activated upon stimulation with agonists of several TLRs. Activated microglia release inflammatory mediators such as nitric oxide (NO) or activin A. Activin A is a member of the transforming growth factor-β (TGF-β) family which plays a central role in different aspects of cell growth and differentiation [24]. Previous studies showed that activin A is increased in the CSF of patients suffering from BM. Furthermore, activin A may decrease the secretion of NO and pro-inflammatory mediators by activated microglia, and enhances microglial proliferation, suggesting that activin A may counteract the activation of microglial cells in BM [25]. The biological functions of members of the TGF-β family are mediated by 2 types of transmembrane serine/threonine kinase receptors (type I and II) that are expressed on activated microglial cells. Therefore, microglia is not only the source but also the target cells of activin A in the CNS [25]. The down-regulation of the *activin A receptor type II*-*like 1* (ID 382555, Figure 4A) upon vitamin B6 treatment may indicate a reduced activation of microglia and thus, a decreased release of tissue-destructive mediators.

G protein coupled receptors (GPCRs) change their conformation upon stimulation by an activating signal. This activated state is controlled by regulators of G protein signaling (RGS) [26,27]. RGS2 (ID 377853) as well as GPCR3 (ID 435019) are up-regulated in vitamin B6-treated rats compared to saline-treated animals, suggesting an involvement of G protein signaling in cellular processes leading to a reduction of hippocampal apoptosis (Figure 4A). Recent studies documented that among the RGS family, RGS2 plays a prominent role in regulating synaptic transmission and plasticity in hippocampal neurons [26]. It is therefore likely that increased RGS2 levels modulate synaptic output, probably leading to an elevated survival of neurons upon vitamin B6 treatment.

The GPCR3 endothelin_B_ (ET_B_) has been found to be important for the physiological reduction of neuronal apoptosis in the dentate gyrus during postnatal development. Furthermore, ET_B_ receptors are involved in pathological apoptosis in the dentate gyrus, especially in a rabbit model of pneumococcal meningitis [28]. These data suggest a role for the ET_B_ receptor as an anti-apoptotic factor in the dentate gyrus, consistent with the up-regulation of *ET*_*B*_*receptor* gene as a result of vitamin B6 treatment.

Another important receptor also belonging to the GPCR superfamily is the neuromedin B receptor (NMBR, ID 438637, Figure 4A). NMBR is localized in brain microvascular endothelial cells that form the blood–brain barrier. It is coupled to the phospholipase C transducing system controlling the K^+^ efflux out of the brain [29]. A down-regulation of NMBR in vitamin B6-treated rats may be indicative for blood–brain barrier disruption induced by BM.

The pro-inflammatory mediator IL-1 consists of 4 molecular species (IL-1α, IL-1β, IL-1γ and IL-1δ) that exert similar biological activity through IL-1 receptor (IL-1R) type I. *In vivo* studies showed that IL-1R^−/−^ mice, lacking an intact IL-1 signal, are more susceptible to develop pneumococcal meningitis, and that the disease is associated with higher mortality in IL-1R^−/−^ mice compared to wild-type animals. These results suggest a protective role of locally produced IL-1 in the first-line defense against pathogens during pneumococcal meningitis [30]. However, in the present study, *IL*-*1R type I* (ID 381937, Figure 4A) was down-regulated, indicating that upon vitamin B6 treatment the host inflammatory reaction is down-modulated.

Highly raised concentrations of CXC-chemokines (e.g. IL-8) that attract neutrophils, and of CC-chemokines (e.g. monocyte chemoattractant protein MCP-1, macrophage inflammatory proteins MIP-1α and MIP-1β) that attract monocytes and lymphocytes, were observed in the CSF during BM [31,32]. Chemokine receptors are also GPCRs. In this study, CCR2 (ID 2818300), the receptor of MCP-1, and CCR5 (ID 382626), the receptor of MIP-1α and MIP-1β, are down-regulated (Figure 4A). Both receptors are expressed on glial and neuronal cells in the adult brain as well as on neural progenitor cells isolated from the subventricular zone where neurogenesis occurs. The localization of chemokine receptors in these regions suggests an involvement of CCR2 and CCR5 in the regulation of adult neural progenitor cells in physiological or pathological conditions [33]. Other studies showed that CCR2 is one of the most prominent chemokine receptor associated with neuro-inflammatory diseases such as multiple sclerosis and experimental autoimmune encephalomyelitis [34]. However, the down-regulation of CCR2 and CCR5 following vitamin B6 treatment may result in a reduced production of neuro-inflammatory mediators by glial or neuronal cells. Furthermore, recruitment of monocytes and lymphocytes to the CSF may also be reduced. Finally, it could also influence the neurogenetic processes observed in the hippocampal dentate gyrus (see below).

Following inflammation, microglial cells become activated and produce inflammatory mediators causing brain damage in a variety of neurodegenerative disorders. Since inflammation may exacerbate brain damage, the control and reduction of brain inflammation is pathophysiologically important. IL-13 is an anti-inflammatory cytokine which minimizes the production of inflammatory mediators from activated microglia (e.g. TNFα and MIP-1α) [35]. Moreover, experimental studies showed that exogenous IL-13 selectively induces apoptotic death of activated microglia [36]. Another study demonstrated that neurons and microglia cooperatively down-regulate brain inflammation by inducing endogenous IL-13 expression in microglia, resulting in microglial death and elevation of neuronal survival [35]. Suggesting a reduced inflammatory reaction as assessed by a down-regulation of pro-inflammatory cytokines (IL-1R type I) and chemokines (CCR2 and CCR5) in vitamin B6-treated rats, the requirement for anti-inflammatory cytokines such as IL-13 is reduced. This suggestion is consistent with the down-modulation of the *IL*-*13 receptor alpha 1* (ID 432716, Figure 4A) gene upon vitamin B6 treatment.

In summary, vitamin B6 down-modulates the inflammatory response as evidenced by reduced RNA levels encoding for pro-inflammatory cytokines and chemokines, and by transcriptional indication for diminished activation of microglia. Because the brain damage observed in BM, including hippocampal apoptosis, is mainly due to the host inflammatory reaction [37], a down-modulated immune reaction may decisively contribute to diminished hippocampal apoptosis observed in vitamin B6-treated rats. Evidence for strong anti-inflammatory effects of vitamin B6 in patients with systemic inflammatory symptoms has also been provided by others [38-40].

### Circadian rhythm

The circadian rhythm is generated by a set of interacting genes and proteins (see Additional file 3: Figure S2). For example in mammals, the protein products of the *clock* and *Bmal1* genes act together to induce the expression of other clock genes including *period* (PER) [41]. The up-regulation of *period homolog* transcripts (PER1, ID 395923, and PER2, ID 392428) in vitamin B6- compared to placebo-treated rats suggests an involvement of the circadian rhythm in the regulation of apoptotic processes (Figure 4B).

Recent studies demonstrated a circadian periodicity of the TRP metabolism via the KYN pathway [42]. However, TRP metabolism in the brain mainly occurs via 2 different pathways, the methoxyindole and the KYN pathway. In experimental models as well as in humans, melatonin, the main metabolite of the methoxyindole pathway, acts as neuroprotective agent. It inhibits the NMDA receptor and thus, protects the neurons from excitotoxic damage. The same effect is mediated by KYNA, a neuroprotective metabolite of the KYN pathway. The inhibition of the NMDA receptor activity partially depends on the reduction of the NO synthase activity, therefore decreasing the amount of NO produced as a result of NMDA activation. Melatonin also follows a circadian rhythmic pattern, mainly determined by the pineal gland that increases the production of melatonin upon physiological stimuli such as darkness. Activation of either the methoxyindole or the KYN pathway reaches an equilibrium in normal conditions by an increase in the TRP degradation via the KYN pathway during the day and via the methoxyindole pathway during the night [43]. This equilibrium is lost under conditions of stress including febrile and epileptic seizures and probably also in other pathological situations [44]. BM displaying a stress situation could influence the equilibrium between the methoxyindole and the KYN pathway. Because vitamin B6 acts as a cofactor for 2 key enzymes of the KYN pathway and also positively affects the pineal production of melatonin [43], administration of vitamin B6 could restore this equilibrium. Therefore, melatonin as a immunomodulatory agent could play an important role in neuroinflammation and subsequent brain injury [45].

The elevation of cellular NAD+ levels through the vitamin B6 induced activation of the KYN pathway observed in this study, may also have an influence on factors involved in the circadian rhythm described above. NAD+ has been shown to act as a central circadian regulator. Concerning the role of NAD+ in cellular energy stores, a molecular coupling between the circadian rhythm and energy metabolism has been proposed [46-48]. Moreover, a link between disruption of circadian rhythm and hippocampal learning and memory has been reported in rats using the water maze task. Chronic stress, sleep deprivation and decreases in melatonin secretion are some of the many side effects of circadian disruption. By its anti-oxidant and neuroprotective role in the brain, melatonin deprivation may contribute to brain damage in individuals suffering from chronic circadian disruption. In transgenic mouse models of Alzheimer’s disease, melatonin treatment may reduce the deposition of β-amyloid and protects against oxidative stress. A possible speculation is that with decreasing levels of melatonin, individuals suffering from chronic circadian disruption become more vulnerable to brain damage associated with learning and memory impairment [49]. Another study showed that the *clock* gene could have an important role on spatial learning in mice, as assessed by water maze [50]. Furthermore, experimental mouse models suggest that cell cycle and apoptotic processes may be regulated by circadian *clock* genes in bone marrow [41].

### Neuronal signaling

Neurogenesis, the continuous production of new neurons from a population of dividing neural progenitor cells, occurs in the hippocampal dentate gyrus. It is influenced by pathological situations such as ischemia or inflammation. BM may affect the production of neuronal survival factors such as *brain*-*derived neurotrophic factor* (BDNF, ID 382982, Figure 4C) gene, thereby promoting the survival of neuronal cells and thus, having an impact on neurogenetic processes [51].

Recent studies demonstrated that the expression of BNDF and its receptor TrkB is increased in mature neurons during the acute phase of pneumococcal meningitis [1]. BDNF protein co-localizes with cells expressing TrkB in the hippocampal CA3/4 region and the hilus adjacent to the subgranular zone of the dentate gyrus where the proliferation of progenitor cells is increased. These findings indicate an involvement of endogenous BDNF and TrkB signaling in neurogenesis after BM [23]. However, the persistence of neurological sequelae in up to 50% of survivors from BM [1,3] suggests that endogenous mechanisms responsible for neuroregeneration are inefficient.

Since treatment with exogenous BDNF results in the reduction of various forms of cell death in experimental pneumococcal meningitis [52], one can speculate that the up-regulated expression level of BDNF in vitamin B6-treated animals plays an important role in diminishing hippocampal apoptosis.

BDNF induces the expression of many genes in hippocampal cells in culture, including *activity regulated cytoskeletal*-*associated protein* (ARC, ID 382171, Figure 4C) gene. ARC itself is involved in memory consolidation and long-term potentiation [53]. Because injury to the hippocampal dentate gyrus is associated with learning and memory deficits [5], the up-regulation of ARC RNA in our study provides further evidence for a role of BDNF in the reduction of hippocampal apoptosis.

Another gene involved in neuronal signaling processes is *early growth response 2* (EGR2, ID 438557, Figure 4C). EGR2 is an important mediator of the growth-suppressive signal of phosphatase and tensin homolog (PTEN) and plays a key role in the PTEN-induced apoptotic pathway. It alters the permeability of mitochondrial membranes, resulting in the release of cytochrome c which in turn activates caspase-3, -8 and −9. As an alternative route, EGR2 may directly induce the expression of pro-apoptotic factors of the Bcl-2 family [54]. In the present study, EGR2 is up-regulated by vitamin B6 treatment. This result is not consistent with a reduction of apoptotic cell death by vitamin B6. This discrepancy between an induction of apoptosis by EGR2 and an up-regulation of EGR2 under circumstances that have been proven to diminish apoptosis may be due to different experimental conditions. In both studies, the molecular mechanisms of the apoptotic pathway were analyzed by microarrays, but we used an *in vivo* model system of BM, whereas cancer-derived cells served as *in vitro* culture system for the study performed by Unoki and Nakamura [54]. Furthermore, posttranslational mechanisms such as phosphorylation, important for the biological activity of PTEN, are not considered in microarray experiments.

Members of the nuclear receptor subfamily 4 group A (NR4A) are classified as early response genes expressed in a wide variety of metabolically demanding and energy dependent tissues such as the brain. They are induced by a broad range of signals, including stress, growth factors, inflammatory cytokines, hormones, calcium, neurotransmitters and physical stimuli. Consistent with the pleiotropic physiological stimuli inducing the NR4A members, these receptors have been implicated in cell cycle regulation, apoptosis, neurological disease, inflammation, carcinogenesis and atherogenesis [55]. Since BM is an inflammatory disease associated with brain damage due to hippocampal apoptosis and often leads to neurological deficits, the NR4A subfamily may play an essential role in this disease. In the present study, both member 1 and 2 of the NR4A family (NR4A1, ID 382110, and NR4A2, ID 438446) are up-regulated, suggesting an involvement in apoptotic processes (Figure 4C). Recent studies showed that the role of the Nr4A members in cancer is largely defined by the implication of the subfamily in the regulation of apoptosis [55]. Furthermore, experimental studies with macrophages demonstrated an involvement of NR4A1 in modulating apoptosis in the inflammatory response. Recent work also suggested that in certain cell lines NR4A1 translocates to the mitochondria to release cytochrome c [56].

### Apoptosis/cell death

Platelet activating factor (PAF) is an extremely potent activator of inflammatory cells owing to the expression of its receptor by numerous cells of the innate immune system. Accordingly, hydrolysis of PAF by extracellular or intracellular PAF acetylhydrolases is predicted to inhibit inflammatory signaling. Indeed, expression of plasma PAF acetylhydrolase is increased by stimulation with inflammatory agonists such as LPS, and decreased by anti-inflammatory drugs [57]. Given the possible anti-inflammatory effect of vitamin B6 as suggested by reduced levels of pro-inflammatory mediators (e.g. cytokines and chemokines) and diminished activation of inflammatory cells (e.g. microglia), vitamin B6 may down-regulate the expression of PAF hydrolase. This hypothesis was tested by the vitamin B6 induced attenuation of *PAF acetylhydrolase 2* (PAFAH2, ID 549642, Figure 4D) levels in our study.

PAF induces apoptosis independent of its receptor, but the mechanism underlying this ability is not fully understood [57]. However, PAFAH2 hydrolyzes not only PAF but also short-chain phospholipids [58]. These substrates are pro-apoptotic, pointing to an essential role of PAFAH2 as anti-apoptotic agent [57]. Recent studies reported that a transfection of the plasma PAFAH2 gene reduces glutamate-induced apoptosis in cultured rat cortical neurons. Moreover, studies using a mouse model of focal cerebral ischemia showed that PAFAH2 exerts strong neuroprotective effects against ischemic injury in the CNS by protecting neurons against oxidative stress [58]. In this context, it seems that down-regulated PAFAH2 does not contribute to the processes leading to the reduced hippocampal apoptosis in vitamin B6-treated rats.

Beside the role of matrix metalloproteinases (MMPs) in blood–brain barrier disruption and extravasation of inflammatory cells into the CNS [3,59], recent studies suggested an involvement of MMPs in glial and neuronal cell death. Furthermore, an excessive increase of MMP-9 (ID 382260, Figure 4D) in BM has been identified as a risk factor for the development of neurological sequelae [59]. Therefore, the down-regulation of MMP-9 upon vitamin B6 treatment indicates a long-term effect of vitamin B6 in terms of reduced learning and memory impairments.

MMPs are also increased by antimicrobial peptides. Antimicrobial peptides are effector molecules of the innate immune system with antibiotic function. Aside from their antibiotic functions, they may be involved in immune responses and inflammatory disease. For example, they may amplify inflammation by activation of cytokine and chemokine expression in immune cells [60]. Lysozyme (Lyz) is an antimicrobial protein belonging to the defensin family of host-defense proteins which are distributed widely in biological fluids and tissues. Experimental studies with transgenic mice showed that Lyz raises the levels of antioxidant reserves that are required to manage non-pathological amounts of reactive oxygen species. These antioxidant properties are partly mediated via negative regulation of stress response genes (e.g. c-Jun) and also involve the blockade of cellular apoptosis *in vitro*[61]. However, Brandenburg *et al*. reported that there is no increase of Lyz in the CSF and serum samples from patients with meningitis [60]. In the present study, we found a down-regulation of Lyz 2 (ID 378051, Figure 4D) in vitamin B6-treated rats when compared to saline-treated animals. This down-regulation could be a further indication of a reduced inflammation and in this context, would explain the reduced levels of pro-inflammatory cytokines and chemokines.

Recent studies showed that adjuvant BDNF protects the brain from caspase 3-dependent hippocampal apoptosis in experimental BM [13]. In the present study, up-regulated endogenous BDNF is also involved in apoptotic processes as indicated by the apoptotic cell death cluster (Figure 4D). This result provides further evidence for a crucial role of BDNF in reducing hippocampal apoptosis upon vitamin B6 treatment.

But how does vitamin B6 induce BDNF expression? Several studies showed that BDNF expression in neuronal cells is induced by activation of calcium channels and recruitment of calcium-sensitive transcription factors [62]. The excitatory amino acid glutamate which is increased in interstitial brain fluid in BM [63] induces a calcium influx by binding to the NMDA receptor and thus, may stimulate the production of BDNF [64]. On the contrary, KYNA, the neuroprotective intermediate of the KYN pathway, is an antagonist of the NMDA receptor and therefore, inhibits calcium influx. Moreover, *in vitro* studies using rat cerebral cortex nerve terminals showed that vitamin B6 inhibits glutamate release through the suppression of calcium influx [65].

However, other studies reported that high levels of IL-1β decrease BDNF mRNA expression in the rat hippocampus [66]. Thus, the increased amount of BDNF transcripts in vitamin B6-treated rats may result from decreased levels of IL-1β. This suggestion is also supported by the down-regulation of the *IL*-*1R type I* gene as discussed previously.

A related phenomenon could be observed in the brains of rats administered an antibiotic plus dexamethasone. Given the up-regulation of BDNF RNA and protein in this study, Li *et al*. hypothesize that the adjuvant therapy with dexamethasone might have a beneficial effect on BM via up-regulation of neuroprotective BDNF. In addition, this study demonstrated a dose-dependent down-regulation of BDNF RNA and protein in rats treated with antibiotics alone. A possible reason for this finding is the lysis of bacteria caused by the antibiotic treatment, resulting in the release of bacterial components that stimulate the expression of pro-inflammatory mediators such as IL-1β [66].

## Conclusions

Pre-treatment with vitamin B6 in BM exerts neuroprotective effects in terms of reduced apoptosis in the hippocampal dentate gyrus of infant rats. Although the processes required for this effect need more investigation, preservation of cellular energy stores, reduction of the inflammatory response and up-regulation of BDNF expression may, at least partially, explain the neuroprotective properties of vitamin B6 in models of pneumococcal meningitis.

## Abbreviations

ARC: Activity regulated cytoskeletal-associated protein; BDNF: Brain-derived neurotrophic factor; BM: Bacterial meningitis; CNS: central nervous system; CSF: Cerebrospinal fluid; DAVID: Database for annotation, visualization and integrated discovery; EGR2: Early growth response 2; ETB: Endothelin_B_; GCOS: GeneChip® operating software; GO: Gene ontology; GPCRs: G protein coupled receptors; IL-1R: IL-1 receptor; i.p.: Intraperitoneal; KEGG: Kyoto Encyclopedia of genes and genomes; KYN: Kynurenine; KYNA: Kynurenic acid; Limma: Linear models for microarray data; LYZ: Lysozyme; MAPK: Mitogen-activated protein kinase; MCP: Monocyte chemoattractant protein; MIP: Macrophage inflammatory protein; MMP: Matrix metalloproteinase; NAD+: Nicotine amide adenine dinucleotide; NMBR: Neuromedin B receptor; NMDA: *N*-methyl-D-aspartate; NO: Nitric oxide; Npas4: Neuronal PAS domain protein 4; NR4A: Nuclear receptor subfamily 4 group A; PAF: Platelet activating factor; PAFAH2: PAF acetylhydrolase 2; PBS: Phosphate buffered saline; PER: Period; PTEN: Phosphatase and tensin homolog; RefSeq: Reference sequences; RGS: Regulators of G protein signaling; RMA: Robust multi average; Rpl24: Ribosomal protein L24; s.c.: Subcutaneously; TGF-β: Transforming growth factor-β; TLRs: Toll-like receptors; TRP: Tryptophan.

## Competing interests

All authors declared that they have no competing interests.

## Authors’ contributions

MW and SLL conceived and designed the study. DCZ and CLB performed the experiments. DCZ and MW analyzed the data. DCZ, MW and SLL wrote the paper. All authors read and approved the final manuscript.

## Pre-publication history

The pre-publication history for this paper can be accessed here:

http://www.biomedcentral.com/1471-2334/13/393/prepub

## Supplementary Material

Additional file 1: Table S1 Gene list. Green marked genes are up-regulated, red marked genes are down-regulated. The genes are arranged according to decreasing fold change. Click here for file

Additional file 2: Figure S1 MAPK signaling pathway. Pathway analysis of significantly regulated genes according to KEGG database. The genes are stained according to their regulation level: Green marked genes are up-regulated, red marked genes are down-regulated and light green stained genes are on control level. Click here for file

Additional file 3: Figure S2 Circadian rhythm. Pathway analysis of significantly regulated genes according to KEGG database. The genes are stained according to their regulation level: Green marked genes are up-regulated, red marked genes are down-regulated and light green stained genes are on control level. Click here for file
