# Supplementary material for: Vitamin B6 reduces hippocampal apoptosis in experimental pneumococcal meningitis
Source: BMC Infect Dis. 2013 Aug 27;13:393. doi: 10.1186/1471-2334-13-393 (PMC3765858; doi:10.1186/1471-2334-13-393)
Supplement: Additional file 1: Table S1 — Gene list. Green marked genes are up-regulated, red marked genes are down-regulated. The genes are arranged according to decreasing fold change. [file 1471-2334-13-393-S1.doc]

| **Affy-ID** | **Gene Description** | **Gene Symbol** | **Fold Change** | **adj.p value** | **Gene Accession** |
| --- | --- | --- | --- | --- | --- |
| 10727717 | neuronal PAS domain protein 4 | Npas4 | 17.783547 | 0.02343367 | NM_153626 |
| 10719432 | FBJ osteosarcoma oncogene B | Fosb | 8.90672222 | 0.01982223 | ENSRNOT00000022556 |
| 10899387 | nuclear receptor subfamily 4, group A, member 1 | Nr4a1 | 5.70495616 | 0.02957022 | NM_024388 |
| 10904511 | activity regulated cytoskeletal-associated protein | Arc | 5.40039974 | 0.02930384 | NM_019361 |
| 10886031 | FBJ osteosarcoma oncogene | Fos | 4.44780395 | 0.01919529 | NM_022197 |
| 10832802 | early growth response 2 | Egr2 | 3.57969185 | 0.01919529 | NM_053633 |
| 10800919 | early growth response 1 | Egr1 | 3.17918198 | 0.02850111 | NM_012551 |
| 10852620 | brain-derived neurotrophic factor | Bdnf | 3.14497555 | 0.03324756 | NM_012513 |
| 10845384 | nuclear receptor subfamily 4, group A, member 2 | Nr4a2 | 2.7477581 | 0.02343367 | NM_019328 |
| 10768332 | regulator of G-protein signaling 2 | Rgs2 | 2.66287702 | 0.0263369 | NM_053453 |
| 10738477 | ADP-ribosylation factor 4-like | Arf4l | 2.45689261 | 0.01982223 | NM_001107052 |
| 10863676 | early growth response 4 | Egr4 | 2.35907421 | 0.0261521 | NM_019137 |
| 10781337 | early growth response 3 | Egr3 | 2.27940297 | 0.0261521 | NM_017086 |
| 10716080 | dual specificity phosphatase 5 | Dusp5 | 2.14888369 | 0.02045727 | NM_133578 |
| 10832197 | SNF1-like kinase | Snf1lk | 1.96723458 | 0.0337849 | NM_021693 |
| 10830908 | ring finger protein 39 | Rnf39 | 1.88652027 | 0.0109708 | NM_134374 |
| 10929842 | period homolog 2 (Drosophila) | Per2 | 1.84231199 | 0.01919529 | NM_031678 |
| 10734882 | period homolog 1 (Drosophila) | Per1 | 1.8289677 | 0.01919529 | NM_001034125 |
| 10797527 | growth arrest and DNA-damage-inducible 45 gamma | Gadd45g | 1.66140039 | 0.03666248 | NM_001077640 |
| 10861986 | insulin induced gene 1 | Insig1 | 1.66030904 | 0.01838103 | NM_022392 |
| 10789653 | insulin receptor substrate 2 | Irs2 | 1.6314489 | 0.0337849 | ENSRNOT00000032918 |
| 10796543 | ADP-ribosylation factor-like 5B | Arl5b | 1.60718321 | 0.0263369 | NM_001015031 |
| 10872626 | G protein-coupled receptor 3 | Gpr3 | 1.58398602 | 0.04216759 | NM_153727 |
| 10719990 | inositol 1,4,5-trisphosphate 3-kinase C | Itpkc | 1.56632002 | 0.0261521 | NM_178094 |
| 10859880 | insulin induced gene 1 | Insig1 | 1.52648943 | 0.0109708 | NM_022392 |
| 10862747 | SPT2, Suppressor of Ty, domain containing 1 (S. cerevisiae) | Spty2d1 | 1.49302694 | 0.03553836 | ENSRNOT00000008420 |
| 10817696 | protein kinase, AMP-activated, beta 2 non-catalytic subunit | Prkab2 | 1.46691452 | 0.01982223 | NM_022627 |
| 10820666 | 3-hydroxy-3-methylglutaryl-Coenzyme A reductase | Hmgcr | 1.44629114 | 0.01958824 | NM_013134 |
| 10787841 | sterol-C4-methyl oxidase-like | Sc4mol | 1.41732833 | 0.02989363 | NM_080886 |
| 10847525 | cryptochrome 2 (photolyase-like) | Cry2 | 1.41729414 | 0.01919529 | NM_133405 |
| 10921550 | serum response factor | Srf | 1.40394167 | 0.03796911 | NM_001109302 |
| 10781927 | similar to 9630044O09Rik protein | RGD1308626 | 1.40114824 | 0.03429793 | BC088867 |
| 10769361 | flavin containing monooxygenase 1 | Fmo1 | 1.39990585 | 0.03213349 | NM_012792 |
| 10838117 | regeneration associated muscle protease | Ramp | 1.3897005 | 0.02973463 | NM_001107755 |
| **Affy-ID** | **Gene Description** | **Gene Symbol** | **Fold Change** | **adj.p value** | **Gene Accession** |
| 10715025 | BTAF1 RNA polymerase II, B-TFIID transcription factor-associated, (Mot1 homolog, S. cerevisiae) | Btaf1 | 1.37445798 | 0.03505765 | ENSRNOT00000054751 |
| 10799241 | isopentenyl-diphosphate delta isomerase | Idi1 | 1.36828086 | 0.02973463 | NM_053539 |
| 10774375 | pellino homolog 1 (Drosophila) | Peli1 | 1.36374565 | 0.03315866 | BC166745 |
| 10855853 | protein phosphatase 1K (PP2C domain containing) | Ppm1k | 1.36252849 | 0.03678195 | NM_001107863 |
| 10711743 | similar to erythroid differentiation-related factor 1 | RGD1306820 | 1.32621861 | 0.03324756 | NM_001107557 |
| 10909411 | ubiquitin specific peptidase 2 | Usp2 | 1.32069104 | 0.04941972 | NM_053774 |
| 10779423 | Fez family zinc finger 2 | Fezf2 | 1.30349279 | 0.02850111 | NM_001107251 |
| 10800726 | mitogen activated protein kinase kinase kinase 2 | Map3k2 | 1.29287733 | 0.04941972 | ENSRNOT00000060996 |
| 10811560 | mevalonate (diphospho) decarboxylase | Mvd | 1.292171 | 0.03324756 | NM_031062 |
| 10769680 | hydroxysteroid (17-beta) dehydrogenase 7 | Hsd17b7 | 1.2414996 | 0.03505765 | NM_017235 |
| 10734045 | leucine rich repeat containing 48 | Lrrc48 | 1.21574006 | 0.0263369 | NM_001013857 |
| 10824926 | phosphatidylinositol-4-phosphate 5-kinase, type 1, alpha | Pip5k1a | 1.19980172 | 0.02957022 | NM_001042621 |
| 10781290 | ectonucleoside triphosphate diphosphohydrolase 4 | Entpd4 | 1.19664365 | 0.0263369 | NM_001108384 |
| 10806913 | ubiquitin specific peptidase 38 | Usp38 | 1.18575981 | 0.04941972 | NM_001107418 |
| 10806829 | protein kinase N1 | Pkn1 | -1.15904068 | 0.04663355 | NM_017175 |
| 10868855 | acidic nuclear phosphoprotein 32 family, member B | Anp32b | -1.1603482 | 0.03282947 | NM_131911 |
| 10742533 | PHD finger protein 15 | Phf15 | -1.16258419 | 0.03653991 | NM_001106998 |
| 10800959 | polyadenylate-binding protein interacting protein 2 | Paip2 | -1.17087332 | 0.04172555 | NM_001014148 |
| 10778552 | similar to calcium-regulated heat-stable protein (24kD) | LOC289806 | -1.17926733 | 0.02973463 | ENSRNOT00000045434 |
| 10917795 | c-src tyrosine kinase | Csk | -1.18910458 | 0.03429793 | NM_001030039 |
| 10810570 | exocyst complex component 3-like | Exoc3l | -1.20248008 | 0.02841311 | NM_001106178 |
| 10926930 | minichromosome maintenance deficient 3 (S. cerevisiae) | Mcm3 | -1.21270614 | 0.03505765 | ENSRNOT00000017081 |
| 10875680 | copine III | Cpne3 | -1.22044679 | 0.03553836 | NM_001107917 |
| 10748382 | karyopherin (importin) alpha 2 | Kpna2 | -1.22908305 | 0.0263369 | NM_053483 |
| 10761311 | tRNA-yW synthesizing protein 1 homolog (S. cerevisiae) | Tyw1 | -1.23095293 | 0.01982223 | NM_001107137 |
| 10918385 | lactamase, beta | Lactb | -1.23330481 | 0.03505765 | NM_001106833 |
| 10861474 | cysteine rich BMP regulator 2 (chordin like) | Crim2 | -1.23429222 | 0.03315866 | ENSRNOT00000050795 |
| 10872790 | platelet-activating factor acetylhydrolase 2 | Pafah2 | -1.23478015 | 0.02144851 | NM_177932 |
| 10701839 | neuromedin B receptor | Nmbr | -1.23669351 | 0.0263369 | NM_012799 |
| 10777209 | leucine aminopeptidase 3 | Lap3 | -1.23879821 | 0.03324756 | NM_001011910 |
| 10914916 | jerky homolog-like (mouse) | Jrkl | -1.23939495 | 0.01982223 | NM_001108122 |
| 10775628 | annexin A3 | Anxa3 | -1.24282658 | 0.04215097 | NM_012823 |
| 10879278 | tyrosine kinase with immunoglobulin-like and EGF-like domains 1 | Tie1 | -1.2457502 | 0.04941972 | BC085911 |
| **Affy-ID** | **Gene Description** | **Gene Symbol** | **Fold Change** | **adj.p value** | **Gene Accession** |
| 10882761 | karyopherin (importin) alpha 2 | Kpna2 | -1.25178396 | 0.02045727 | NM_053483 |
| 10708672 | prolylcarboxypeptidase (angiotensinase C) | Prcp | -1.25341017 | 0.02973463 | NM_001106281 |
| 10856797 | dysferlin | Dysf | -1.26319941 | 0.02045727 | NM_001107869 |
| 10894306 | similar to a disintegrin-like and metalloprotease (reprolysin type) with thrombospondin type 1 motif, 10 | LOC314655 | -1.26490041 | 0.0263369 | BC101863 |
| 10746976 | topoisomerase (DNA) II alpha | Top2a | -1.26884074 | 0.0337849 | NM_022183 |
| 10741970 | dedicator of cytokinesis 2 | Dock2 | -1.2767611 | 0.0263369 | BC101877 |
| 10872015 | similar to 2610027C15Rik protein | RGD1308876 | -1.3096125 | 0.01919529 | BC167091 |
| 10810144 | ribonucleotide reductase M2 | Rrm2 | -1.31007536 | 0.03282947 | NM_001025740 |
| 10901823 | thymopoietin | Tmpo | -1.32275842 | 0.02973463 | NM_012887 |
| 10736545 | nucleoredoxin | Nxn | -1.32423576 | 0.04188704 | NM_001108285 |
| 10771826 | ADAM metallopeptidase with thrombospondin type 1, motif 3 | Adamts3 | -1.32695808 | 0.04188704 | NM_001107212 |
| 10863512 | similar to Bifunctional methylenetetrahydrofolate dehydrogenase/cyclohydrolase, mitochondrial precursor | LOC680308 | -1.3316639 | 0.02850111 | NM_001109398 |
| 10776676 | shisa homolog 3 (Xenopus laevis) | Shisa3 | -1.3383416 | 0.0337849 | NM_001109087 |
| 10806129 | minichromosome maintenance deficient 5, cell division cycle 46 (S. cerevisiae) | Mcm5 | -1.34027477 | 0.0261521 | NM_001106170 |
| 10864279 | leucine-rich repeats and immunoglobulin-like domains 1 | Lrig1 | -1.34946803 | 0.04941972 | ENSRNOT00000017384 |
| 10869303 | solute carrier family 31, member 2 | Slc31a2 | -1.35190053 | 0.0263369 | NM_001033693 |
| 10818090 | solute carrier family 16 (monocarboxylic acid transporters), member 1 | Slc16a1 | -1.35191533 | 0.0263369 | NM_012716 |
| 10881293 | podoplanin | Pdpn | -1.35273196 | 0.01919529 | NM_019358 |
| 10909263 | similar to Discs, large homolog 5 (Placenta and prostate DLG) | RGD1563738 | -1.3567151 | 0.01919529 | ENSRNOT00000043957 |
| 10916264 | similar to Discs, large homolog 5 (Placenta and prostate DLG) | RGD1563738 | -1.35815901 | 0.04188704 | ENSRNOT00000043957 |
| 10883903 | ribonucleotide reductase M2 | Rrm2 | -1.35864618 | 0.01919529 | NM_001025740 |
| 10930428 | elastin microfibril interfacer 2 | Emilin2 | -1.3800734 | 0.03213349 | ENSRNOT00000019937 |
| 10789857 | interleukin 17 receptor B | Il17rb | -1.38212529 | 0.01919529 | NM_001107290 |
| 10897465 | pleckstrin homology, Sec7 and coiled/coil domains 4 | Pscd4 | -1.3868833 | 0.0261521 | ENSRNOT00000010413 |
| 10899354 | activin A receptor type II-like 1 | Acvrl1 | -1.38693714 | 0.02860102 | NM_022441 |
| 10912614 | solute carrier organic anion transporter family, member 2a1 | Slco2a1 | -1.4051033 | 0.0337849 | NM_022667 |
| 10824123 | kin of IRRE like (Drosophila) | Kirrel | -1.40696243 | 0.03448334 | NM_207606 |
| 10868627 | GLI pathogenesis-related 2 | Glipr2 | -1.41218569 | 0.01919529 | ENSRNOT00000019916 |
| 10811596 | family with sequence similarity 38, member A | Fam38a | -1.41722184 | 0.02045727 | NM_001077200 |
| 10847761 | CD44 antigen | Cd44 | -1.42621512 | 0.04215097 | NM_012924 |
| 10786401 | wingless-related MMTV integration site 5A | Wnt5a | -1.43879654 | 0.0263369 | NM_022631 |
| 10765772 | transgelin 2 | Tagln2 | -1.4454 | 0.03282947 | NM_001013127 |
| 10831090 | chloride intracellular channel 1 | Clic1 | -1.47105542 | 0.0337849 | NM_001002807 |
| **Affy-ID** | **Gene Description** | **Gene Symbol** | **Fold Change** | **adj.p value** | **Gene Accession** |
| 10825153 | Fc receptor, IgG, high affinity I | Fcgr1 | -1.476463 | 0.03213349 | NM_001100836 |
| 10914618 | chemokine (C-C motif) receptor 5 | Ccr5 | -1.477582 | 0.03728695 | NM_053960 |
| 10723884 | serine (or cysteine) peptidase inhibitor, clade H, member 1 | Serpinh1 | -1.48327243 | 0.0263369 | NM_017173 |
| 10812775 | CD180 molecule | Cd180 | -1.53734568 | 0.02930384 | NM_001106405 |
| 10754983 | interleukin 13 receptor, alpha 1 | Il13ra1 | -1.55538292 | 0.02045727 | NM_145789 |
| 10894333 | myosin IF | Myo1f | -1.55578316 | 0.02973463 | NM_001108076 |
| 10922826 | interleukin 1 receptor, type I | Il1r1 | -1.55646787 | 0.03282947 | NM_013123 |
| 10888404 | RAS, guanyl releasing protein 3 | Rasgrp3 | -1.55753005 | 0.02878092 | NM_001108009 |
| 10719204 | EH-domain containing 2 | Ehd2 | -1.56020608 | 0.01919529 | NM_001024897 |
| 10895585 | similar to Lysozyme C type 2 precursor (1,4-beta-N-acetylmuramidase C) | rCG_48647 | -1.58157372 | 0.01919529 | NM_001128494 |
| 10936365 | interleukin 13 receptor, alpha 1 | Il13ra1 | -1.58632349 | 0.0261521 | NM_145789 |
| 10874198 | tumor necrosis factor receptor superfamily, member 9 | Tnfrsf9 | -1.63286939 | 0.03213349 | NM_001025773 |
| 10914614 | chemokine (C-C motif) receptor 2 | Ccr2 | -1.69209433 | 0.02089752 | NM_021866 |
| 10764069 | chitinase 3-like 1 | Chi3l1 | -1.77270688 | 0.02930384 | NM_053560 |
| 10844005 | ficolin B | Fcnb | -1.77791876 | 0.01919529 | NM_053634 |
| 10713833 | RAB3A interacting protein (rabin3)-like 1 | Rab3il1 | -1.77972631 | 0.01919529 | NM_134411 |
| 10888424 | latent transforming growth factor beta binding protein 1 | Ltbp1 | -1.8353075 | 0.01919529 | NM_021587 |
| 10842239 | matrix metallopeptidase 9 | Mmp9 | -1.84655368 | 0.04941972 | NM_031055 |
| 10904578 | Ly6-C antigen | Ly6c | -1.90536712 | 0.03600922 | NM_020103 |
| 10902547 | lysozyme 2 | Lyz2 | -1.9607481 | 0.0263369 | NM_012771 |
