# Supplementary figures and images for: Vitamin B6 reduces hippocampal apoptosis in experimental pneumococcal meningitis
Source: BMC Infect Dis. 2013 Aug 27;13:393. doi: 10.1186/1471-2334-13-393 (PMC3765858; doi:10.1186/1471-2334-13-393)

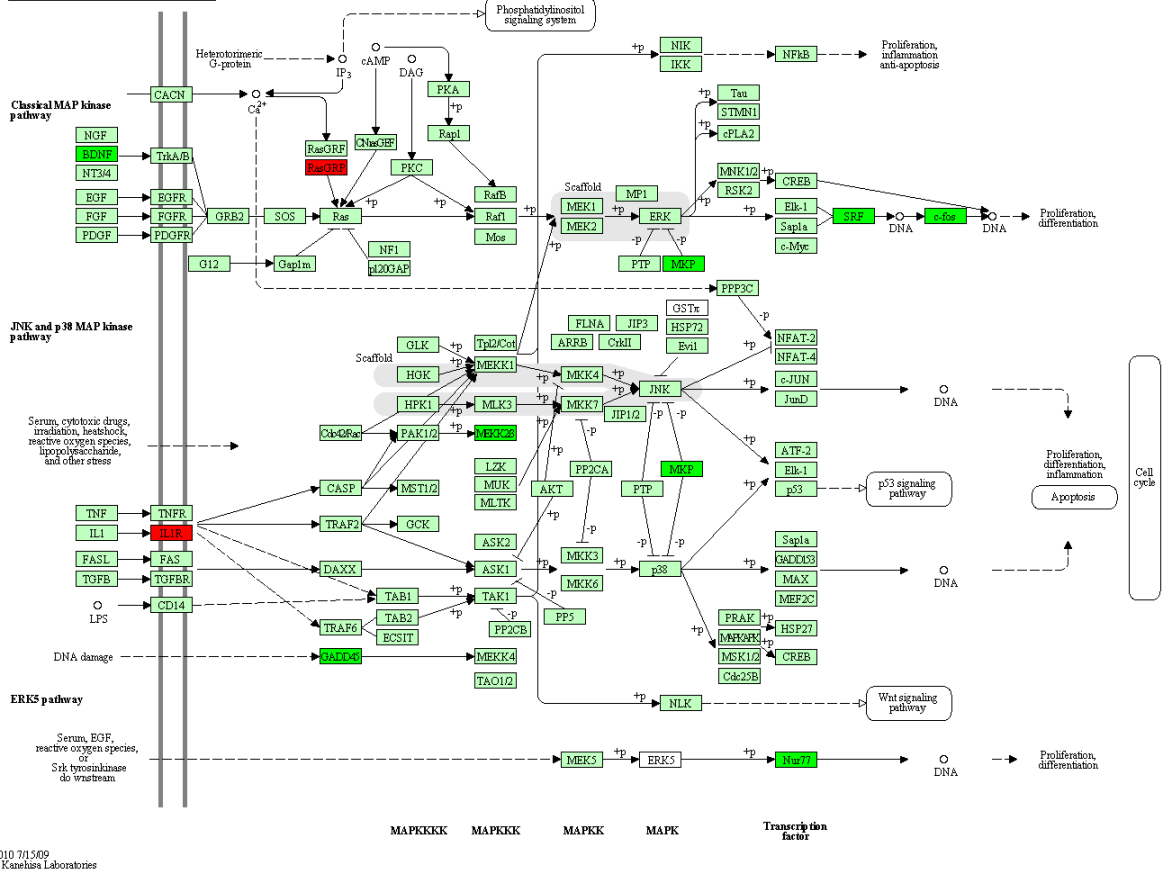

Supplement: Additional file 2: Figure S1 — MAPK signaling pathway. Pathway analysis of significantly regulated genes according to KEGG database. The genes are stained according to their regulation level: Green marked genes are up-regulated, red marked genes are down-regulated and light green stained genes are on control level. [file 1471-2334-13-393-S2.pdf]

# CIRCADIAN RHYTHM

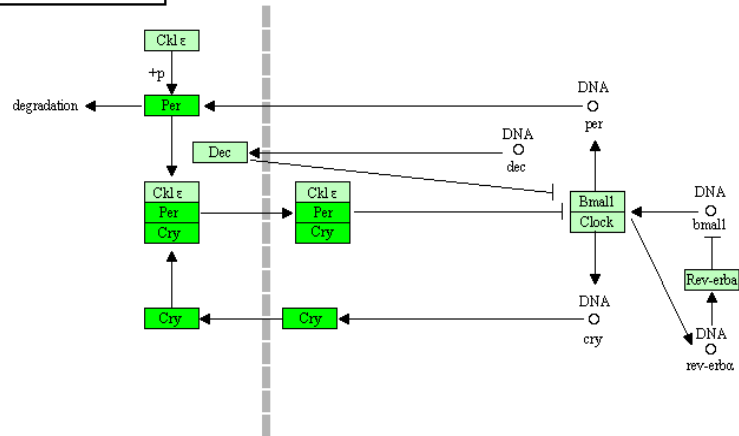

Supplement: Additional file 3: Figure S2 — Circadian rhythm. Pathway analysis of significantly regulated genes according to KEGG database. The genes are stained according to their regulation level: Green marked genes are up-regulated, red marked genes are down-regulated and light green stained genes are on control level. [file 1471-2334-13-393-S3.pdf]
